# Mentalizing in an economic games context is associated with enhanced activation and connectivity in the left temporoparietal junction

**DOI:** 10.1093/scan/nsad023

**Published:** 2023-05-02

**Authors:** Li-Ang Chang, Konstantinos Armaos, Lotte Warns, Ava Q Ma de Sousa, Femke Paauwe, Christin Scholz, Jan B Engelmann

**Affiliations:** Center for Research in Experimental Economics and Political Decision Making (CREED), Amsterdam School of Economics, University of Amsterdam, Roeterstraat 11, Amsterdam 1018WB, The Netherlands; Faculty of Business and Economics, University of Lausanne, Lausanne CH-1015, Switzerland; Brain and Cognitive Sciences, Institute for Interdisciplinary Studies, University of Amsterdam, Amsterdam 1098 XH, The Netherlands; Brain and Cognitive Sciences, Institute for Interdisciplinary Studies, University of Amsterdam, Amsterdam 1098 XH, The Netherlands; Department of Psychological and Brain Sciences, University of California Santa Barbara, Santa Barbara 93106-9660, USA; Brain and Cognitive Sciences, Institute for Interdisciplinary Studies, University of Amsterdam, Amsterdam 1098 XH, The Netherlands; Amsterdam School of Communication Research, University of Amsterdam, Amsterdam 1018 WV, The Netherlands; Center for Research in Experimental Economics and Political Decision Making (CREED), Amsterdam School of Economics, University of Amsterdam, Roeterstraat 11, Amsterdam 1018WB, The Netherlands; Behavioral Economics, The Tinbergen Institute, Amsterdam 1082 MS, The Netherlands

**Keywords:** trust game, ultimatum game, false-belief task, mentalizing, fMRI, temporoparietal junction, dmPFC, PPI

## Abstract

Prior studies in Social Neuroeconomics have consistently reported activation in social cognition regions during interactive economic games, suggesting mentalizing during economic choice. Such mentalizing occurs during active participation in the game, as well as during passive observation of others’ interactions. We designed a novel version of the classic false-belief task (FBT) in which participants read vignettes about interactions between agents in the ultimatum and trust games and were subsequently asked to infer the agents’ beliefs. We compared activation patterns during the economic games FBT to those during the classic FBT using conjunction analyses. We find significant overlap in the left temporoparietal junction (TPJ) and dorsal medial prefrontal cortex, as well as the temporal pole (TP) during two task phases: belief formation and belief inference. Moreover, generalized Psychophysiological Interaction (gPPI) analyses show that during belief formation, the right TPJ is a target of both the left TPJ and the right TP seed regions, whereas during belief inferences all seed regions show interconnectivity with each other. These results indicate that across different task types and phases, mentalizing is associated with activation and connectivity across central nodes of the social cognition network. Importantly, this is the case for both the novel economic games and the classic FBTs.

## Introduction

Inferring others’ mental states and predicting their intentions and beliefs is a social cognitive ability that supports social interactions. This ability is commonly referred to as ‘theory of mind’ or ‘mentalizing’. Studies in social neuroscience have gathered substantial amounts of data on the neural networks involved in inferring others’ beliefs and intentions. This has yielded multiple meta-analyses with well over 100 studies that jointly have identified consistent activations in a specific brain network (Amodio and Frith, 2006; Decety and Lamm, 2007; Mitchell, 2009; Van Overwalle, 2009; Mar, 2011; Schurz *et al.*, 2014; Molenberghs *et al.*, 2016). The core mentalizing network identified by these studies consists of bilateral temporoparietal junction (TPJ), medial Prefrontal Cortex (mPFC), superior temporal sulcus (STS), temporal pole (TP) and precuneus (sometimes including posterior cingulate cortex [PCC]).

Social neuroeconomics is another strand of research that has progressed relatively independently and that has repeatedly identified activation patterns within a similar network of brain regions when participants decide whether to cooperate with strangers in the context of economic games (for meta-analyses, see Feng *et al.*, 2014; Schurz *et al.*, 2014; Bellucci *et al.*, 2017). The striking overlap of activations when participants perform classic false-belief tasks (FBTs) designed to study basic mentalizing processes and when they make decisions in the context of economic games (see Supplementary Figure S1 for a neurosynth meta-analysis results that illustrates this overlap) has been taken to suggest that participants engage in belief-based inferences that rely on mentalizing about their interaction partners when making interactive economic decisions (Fehr and Camerer, 2007; Alós-Ferrer and Farolfi, 2019; Engelmann *et al.*, 2019). Neuroimaging studies have consistently revealed such social cognitive activations during social decision-making in the context of the trust game (TG) (McCabe *et al.*, 2001; Krueger *et al.*, 2007, 2008; Sripada *et al.*, 2009; Stanley *et al.*, 2012; Engelmann *et al.*, 2019). Similar social cognitive activations have also been observed during the ultimatum and prisoner’s dilemma games (Rilling *et al.*, 2004; Fukui *et al.*, 2006; for a detailed description of these games, see Engemann *et al.*, 2012). The results from an initial study on the neural correlates of trust decisions demonstrated the activation of the dorsal medial prefrontal cortex (dmPFC) during social *vs* non-social interactions between cooperative players (McCabe *et al.*, 2001). This involvement of social cognition regions during trust decisions has been replicated and extended in subsequent studies, which also show recruitment of a wider social cognition network that includes dmPFC, TPJ and STS across different experimental contexts (Krueger *et al.*, 2007, 2008; Sripada *et al.*, 2009; Stanley *et al.*, 2012; Engelmann *et al.*, 2019). In fact, a recent study identified a wider network of regions consisting of the dmPFC, anterior insula (AI) and posterior superior temporal sulcus (pSTS) that is more strongly interconnected with the left temporoparietal junction during trust decisions and in people who are more trusting on average (Engelmann *et al.*, 2019). The trends reflected in these findings are supported by a recent meta-analysis by Feng *et al.* (2014) that shows activations in the precuneus, dmPFC and STS when participants consider unfair (relative to fair) offers.

The notion that the activation of social cognition regions during interactive economic games reflects mentalizing is further supported by theoretical considerations (Fehr and Camerer, 2007; Rilling and Sanfey, 2011; Alós-Ferrer and Farolfi, 2019; Engelmann *et al.*, 2019). In economic games, mutual cooperation typically leads to financial gains for both interaction partners. However, there is a flip side in which financial losses can occur if one interaction partner decides to act selfishly to obtain higher payouts for himself or herself at the cost of the other (Engelmann and Fehr, 2017). Because of the possibility of non-cooperation by their interaction partners and the resulting financial loss, participants have a strong incentive to assess how likely their partners are to reciprocate (Bohnet and Zeckhauser, 2004; Bohnet *et al.*, 2008; Aimone *et al.*, 2014). One way to assess the likelihood of non-cooperation is by taking the perspective of the interaction partner, i.e. via mentalizing, which allows the participant to simulate how an interaction partner might act, given the rules of the game. Activations in social cognition regions at the time point at which participants decide whether to invest an amount of money into another person, therefore, likely reflect mentalizing to assess the degree of strategic uncertainty in a given context, and whether it is worth taking this social risk.

In real life, interactions with others are commonly repeated, and the history of interactions can be used to make inferences about others’ trustworthiness. Another central type of social cognitive process, therefore, takes place in the context of repeated interactions, namely learning about people’s trustworthiness (Krueger *et al.*, 2008; Sladky *et al.*, 2021; Bellucci and Dreher, 2022). To model these types of situations, researchers have employed repeated experimental games in which participants learn about the trustworthiness of interaction partners over the course of multiple trials. In such games, feedback about partners’ decisions activates social cognition regions in the dmPFC, TPJ and PCC (Rilling *et al.*, 2004). More specifically, while the dmPFC is more active during the early stages of trust building, it is relatively less implicated once trust has been established in the later stages of repeated TGs (Krueger *et al.*, 2007). In fact, learning about the characteristics of interaction partners has repeatedly been associated with prediction error signals not just in typical dopaminergic regions (Delgado *et al.*, 2005; King-Casas *et al.*, 2005; Diaconescu *et al.*, 2017) but also in an extended network that includes central social cognition regions (Behrens *et al.*, 2008). A recent neuroimaging study confirms these initial results, showing the presence of social prediction error-like signals in the ventromedial prefrontal cortex and TPJ (Bellucci *et al.*, 2019). Jointly, these results directly implicate key regions within the social cognition network in learning about the anti- *vs* pro-social characteristics of current interaction partners.

Taken together, there is thus considerable evidence suggesting that the overlap of activations in the mentalizing network during FBTs and economic games is related to social cognitive processes involved in assessing and learning about the intentions of interaction partners. An important shortcoming of research in Social Neuroeconomics is that evidence for evoking mentalizing during social decision-making is intermixed with strategic considerations by the players who are likely trying to maximize their utility in the context of economic games. In fact, if the participant is directly involved in the economic interaction, it can be difficult to disentangle social cognitive processes from other cognitive and affective processes involved in social choice (Krueger *et al.*, 2007). It is therefore unclear whether the activation patterns observed during social decisions in economic games reflect mentalizing or other important processes (e.g. reward maximization, strategic considerations and social preferences) that support choice. A potential solution to this problem comes from the literature on third-party observers of economic games (Bellucci *et al.*, 2020). In these paradigms, two players participate in an economic game, and a third party observes the interaction between the two players and can punish players who deviate from a social norm. To decide whether one of the players deserves punishment, the observer has to understand the potential strategies, interaction outcomes and the agents’ intentions. At a neural level, three main networks have been implicated in the affective and cognitive processes involved in third-party punishment (TPP): the salience, default mode and central executive network, with respective key regions in the AI, mPFC and TPJ, as well as the dorsolateral PFC (Krueger and Hoffman, 2016). A recent meta-analysis showed that while both TPP and second-party punishment (SPP) consistently recruit social cognition regions, a clear difference also emerged with TPP more robustly recruiting social cognitive regions (e.g. left TPJ), while SPP preferentially engaged social affective regions, specifically the AI (Bellucci *et al.*, 2020). Even though the TPP paradigm alleviates the problem of simultaneous strategic and mentalizing processes, e.g. by reducing the emotional engagement in punishment (van’t Wout *et al.*, 2006), it is not fully immune to it. Indeed, the decision to punish or not relies upon the integration of inferring the intentions of the players, fairness considerations given the judge’s interpretation of social norms and the willingness to engage in costly punishment. Therefore, multiple cognitive processes come together during TPP that might purely distort social cognitive inferences.

The current experiment addresses these limitations by combining the approaches developed by the two research streams of social neuroscience and social neuroeconomics. Our approach minimizes the distortionary influences of strategic considerations and learning present in economic games, while at the same time requiring our participants to make inferences about interaction partners’ mental states from the point of view of a third-party observer. Specifically, we developed a novel FBT that required participants to apply the rules of two well-established economic games, the TG and ultimatum game (UG), to be able to correctly answer incentivized questions that assessed our participants’ understanding of economic game interactions. In this economic game version of the FBT, participants first read about an interaction between two parties and were then asked to either infer the false belief of one of the interaction partners in one condition or calculate the payoff for one of the interaction partners in another condition. The false belief condition assessed our participants’ understanding of how different economic game situations might cause false beliefs held by one of the interaction partners, while the Outcome condition allowed us to assess our participants’ understanding of the rules of the game and how payouts were computed. The former clearly requires mentalizing, while the latter does not. Our approach, therefore, enabled us to assess belief-based inferences in the context of economic games and compare the activation patterns during belief-based inferences in the context of economic games to those during the standard FBT. Of note, using this approach in which our participants act as observers of economic games between two other agents and form beliefs based on their interactions has the distinct advantage that our subjects’ mentalizing processes are not distorted by the cognitive and affective processes that occur in direct interactions within social dilemmas, or by observers who are responsible for punishing norm digressions (Bellucci *et al.*, 2020). Our approach therefore controls for the distortionary influences of valuation processes, strategic considerations, reputation concerns, fairness considerations and other social preferences, as well as affective reactions that are common to first-person TG and UG interactions, thereby allowing us to identify mentalizing processes in the context of economic games.

Given the strong suggestion from theoretical considerations, prior research and meta-analyses, we expected that belief-based inferences (relative to outcome-based inferences) in the context of economic games lead to similar activation patterns within the mentalizing network as the standard FBT. Moreover, if activation patterns across the two versions of the task are indeed similar, activity within key regions may also be similarly interconnected across the two contexts. Thus, we also assessed the functional connectivity of the mentalizing network averaged across the two task contexts.

## Materials and methods

### Participants

Two pilot studies were conducted to develop and further titrate the novel game-theoretic vignettes. Pilot experiment 1 was conducted online via Qualtrics with 50 participants (33 females, age mean = 33.4 years, s.d. = 8 years) that were recruited via Prolific. Pilot experiment 2 was conducted at the Center for Research in Experimental Economics and Political Decision Making (CREED) with 38 participants (26 females, age mean = 21.9 years, s.d. = 1.9 years). All procedures for pilot experiments were approved by the Economics and Business Ethics Committee at the University of Amsterdam.

Thirty-nine right-handed volunteers participated in the main functional Magnetic Resonance Imaging (fMRI) experiment (18 males, aged 18–33 years, mean (s.d.) = 22.51 (4.03) years) mainly recruited from the participant pool of the Behavioural Science Lab of the Faculty of Social and Behavioral Sciences at the University of Amsterdam (LAB, https://www.lab.uva.nl/lab/home). All participants first underwent an initial screening, which required that participants (1) were between 18 and 40 years of age, 2) were right-handed, 3) had no history of any neurological or mental illness, 4) were fluent in English, 5) agreed to receive mild electric shocks during the experiment, 6) never participated in a corresponding behavioral pilot study previously conducted as part of this study and 7) fulfilled all MRI safety requirements according to the guidelines of Spinoza Center of the University of Amsterdam. Two participants were excluded from further analyses: one due to excessive head movements (>2 × voxel size (6 mm)) and another due to low accuracy of responses (mean accuracy < 3 (s.d.) of sample mean). The final dataset for fMRI analysis, therefore, consisted of 37 participants. Written informed consent was obtained from all participants before their participation. All procedures were implemented in compliance with the guidelines formulated by the Ethics Review Board of the Faculty of Social and Behavioral Sciences, the University of Amsterdam.

### Pilot experiments

We first developed a set of game-theoretic vignettes by outlining a number of interaction scenarios from economic games that reflect the false beliefs of one of the interaction partners. In these scenarios, we built upon two well-established economic games, the TG and the UG, which can be easily explained to participants (see the ‘Vignette stimuli section’ for a detailed description of the novel scenarios, and our project page on osf.io for detailed instructions: https://osf.io/3eg56/?view_only=face48878dd144848d26f1c7d3c47d31). The aim of an initial pilot study that was conducted online via Prolific was to test participants’ understanding of the different vignettes and to identify potential outlier scenarios that might not be easily understood by our participants. Vignettes and subsequent questions that probed participants’ understanding were presented to participants via Qualtrics, and reaction times were recorded. The response times indicated that TG outcome vignettes were perceived as too difficult among the four conditions included in this pilot study (TG outcome average reaction time (RT) = 24.68 s, SE = 1.69; UG outcome average mean RT = 15.80 s, SE = 0.84; TG belief average RT = 15.13 s, SE = 1.00; UG belief average RT = 17.17, SE = 0.89). Because paired *t*-tests showed significantly longer RTs in the TG outcome condition compared to all other conditions (UG outcome, *t*(49) = 6.79, *P *= 1.76 x 10^−8^; TG belief, *t*(49) = 6.37, *P* = 6.24 x 10^−8^; UG belief, *t*(49) = 4.82, *P* = 1.43 x 10^−5^), we simplified the computations required for correct responses by restricting possible answers to multiples of five in the TG outcome scenarios.

Next, we validated our new stimulus set by conducting an additional behavioral pilot in the CREED laboratory. This experiment allowed further fine-tuning of the final set of vignettes and experimental parameters such as the appropriate difficulty and timing of stimuli. The experimental design was equivalent to the design reported in the ‘fMRI Experiment’ section, except that participants were also required to indicate when they completed reading during the vignette period by pressing the space bar. While participants were reminded of this in the instructions, we received a relatively low response rate (32% of all trials), indicating that participants had difficulties with the dual task of reading and button pressing within the given period of time. Given these difficulties and to allow participants to fully concentrate on reading the vignettes and to avoid confusion during the fMRI experiment, button presses were no more required during the vignette period in the fMRI experiment. Participants were paid on a piece rate basis (20c per correct answer) and received an average of 28.38 Euros for their participation (average piece rate earnings of 18.38 plus 10 Euros for completing the online survey). Accuracy and response time results from the pilot study are reported alongside the results from the main fMRI experiment in Tables 1 and 2.

**Table 1.** ANOVA tables for accuracy for three different models that include the fMRI, pilot and combined datasets. Models use a restricted random effects structure with random slopes for the Task Domain factor (except for the pilot model) and random intercepts and were estimated using the AFEX package. ANOVA results are based on logistic regressions with correct/incorrect responses as dependent variable

	fMRI Model		Pilot Model		Combined Model	
	***X^2^***	**Pr(>*X^2^*)**	***X^2^***	**Pr(>*X^2^*)**	***X^2^***	**Pr(>*X^2^*)**
Belief	16.8337	<0.001^***^	6.1234	0.01334	22.7888	<0.001^***^
Domain	0.0312	0.8597	5.4224	0.01988^*^	0.2451	0.620529
Belief X Domain	17.853	<0.001^***^	1.025	0.31134*	14.0474	<0.001^***^
Exp Type				0.4482	0.503188	
Threat	2.392	0.122	6.2523	0.0124^*^	0.4236	0.515137
AIC	1169		1158.6		2333.8	
Observation	3530 (37)		3635 (38)		7165 (75)	
Max VIF	1.11		1.08		1.08	

Akaike Information Criterion (AIC) and the maximal Variance Inflation Factor (Max VIF) are reported in the table. * p<0.05 ** p <0.01 ***p<0.001.

**Table 2.** ANOVA tables for log RT for three different models that include the fMRI, pilot and combined datasets. All models use a maximal random effects structure with random slopes and intercepts and were estimated using the AFEX package. Dependent variable is the logarithm of RT for correct trials only

	fMRI Model		Pilot Model		Combined Model	
	***X^2^***	**Pr(>*X^2^*)**	***X^2^***	**Pr(>*X^2^*)**	***X^2^***	**Pr(>*X^2^*)**
Belief	4.5211	0.03348^*^	2.5879	0.1077	6.7356	0.0094508^**^
Domain	63.9953	<0.001^***^	69.3326	<0.001^***^	132.7936	<0.001^***^
Belief x Domain	69.5061	<0.001^***^	54.4388	<0.001^***^	122.0535	<0.001^***^
ExpType					14.0033	0.0001825^***^
Threat	0.5059	0.4769	1.2318	0.2671	1.6949	0.1929561
Observations	3381 (37)		3492 (38)		6873 (75)	
AIC	2925.5		4062.7		7052.4	
Max VIF	1		1		1	

Akaike Information Criterion (AIC) and the maximal Variance Inflation Factor (Max VIF) are reported in the table. * p<0.05 ** p <0.01 ***p<0.001.

### fMRI experiment

#### Procedure

Participants were first invited to complete an online prescreening questionnaire and a battery of personality measures via Qualtrics before the main fMRI experiment. Participants were given 14 Euros for completing this online survey. In part two of the experiment, participants were invited to the fMRI laboratory at the Behavioral Science Lab of the University of Amsterdam. They were asked to carefully read detailed instructions and complete a quiz afterward to ensure that they fully understood the task, especially the rationale behind the economic games (TG and UG; for instructions, see our project page on osf.io https://osf.io/3eg56/?view_only=face48878dd144848d26f1c7d3c47d31). They were allowed to ask questions during the instructions and the quiz, which the experimenters answered carefully. Moreover, if they provided an incorrect answer for one or more questions (out of a total of eight), their answers were discussed, and the relevant part of the experiment was explained again by the experimenter. In addition, participants had the opportunity to practice the task before the start of the fMRI experiment and completed 12 practice trials. To further ensure the participants’ comprehension of the task, all participants were required to achieve at least 66% accuracy before proceeding to the main experiment. Among all participants, only three required two practice runs, after which they passed the threshold of correct answers. After being placed in the scanner, participants underwent a short button training task to allow familiarization with the button box. Subsequently, they completed four fMRI runs, with each run consisting of 24 trials that were subdivided into 8 blocks of 3 trials each. Participants also underwent electrical stimulation calibration before the first and third runs (for details, see Engelmann *et al.*, 2019) to determine pain thresholds for the Threat condition, which we control for, but do not specifically analyze in the current set of analyses. After scanning, participants filled out an exit questionnaire, after which they were paid their show-up fee and performance bonus.

#### Vignette stimuli

**Fig. 1.**
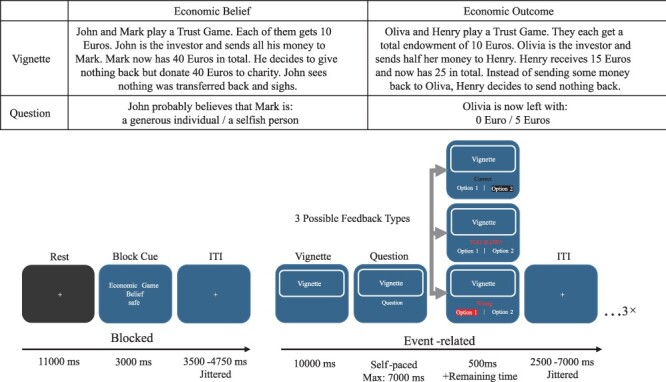
Example Economic Game Vignettes and Task Schematic. (A) A set of novel vignettes based on economic games were developed for the current experiment. The examples in (A) show economic game vignettes in the Belief (left) and the Outcome (right) conditions, together with their respective questions. (B) Trial sequence of the fMRI experiment. An initial block cue indicated the conditions that remained stable for the duration of one block of three trials, including the domain of the vignette, and whether the vignette concerns beliefs or outcomes. The vignette (see A) was shown for 10 s, after which participants were given a maximum of 7 s to answer the question. The correct answers were incentivized at a piece rate of 0.2 Euro.

A novel set of vignettes was developed for the current study, with the aim to test the neural correlates of belief formation and inferences in the context of economic games. These were combined with vignettes from prior research (Saxe and Kanwisher, 2003; Bruneau *et al.*, 2012), to enable comparisons with the well-established FBT. The novel economic game vignettes described interactions between agents in the TG and UG and therefore required an understanding of the rules of these games, which were explained in detailed instructions. Economic game scenarios were based on six different hypothetical events that can occur in laboratory contexts. Importantly, in all scenarios, one interaction partner keeps all, or the majority of the accumulated money for different reasons. The reasons included the participant’s decision to invest their winnings into charity, and incorrect decisions due to a computer error, accidentally pressing the wrong button, or because of misunderstanding the game set-up. Example vignettes are shown in Figure 1A, and the complete list of economic game vignettes can be found on our project page on osf.io (https://osf.io/3eg56/?view_only=face48878dd144848d26f1c7d3c47d31). Additionally, two types of questions were developed that probed participants’ understanding of the interactions described in the vignettes: one type focused on the false belief of one of the agents, while the other type focused on the payouts for one of the agents.

**Fig. 2.**
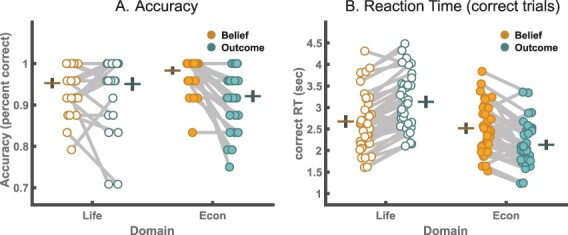
Behavioral results. (A) The mean accuracy across (lines with standard error bounds) and within individuals (connected dots) of participants’ answers (percent correct) across the Domain and Outcome conditions. Accuracy reflects the proportion of correct relative to all responses. (B) The mean response times across (lines with standard error bounds) and within individuals (connected dots) for correct trials only across the Domain and Outcome conditions.

**Fig. 3.**
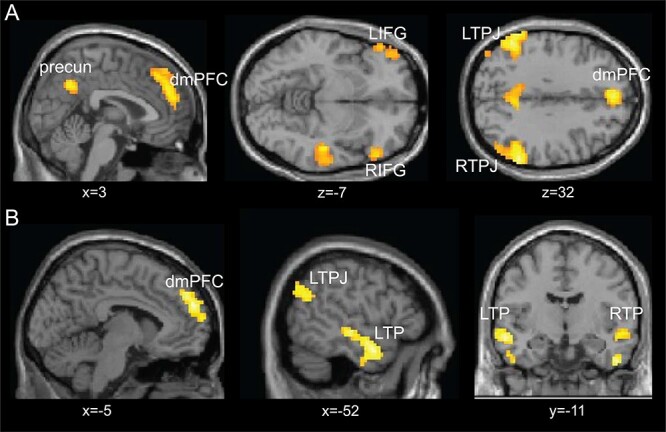
Whole-brain analysis of belief activations during the vignette period for the contrast belief > outcome in the life story domain (A) and economic game domain (B). The results show consistent activations in mentalizing regions in both tasks, particularly in the dmPFC and the left TPJ. The Results shown here were FWE-corrected at the cluster level with a cluster-forming threshold of *P* < 0.001.

Given the novelty of the task, we also assessed whether our participants used a strategy to answer questions about economic game interactions in an open-ended question that was included in the exit questionnaire. We find that a subset of participants indeed used an identifyable strategy to answer questions about economic-game vignettes. We, therefore, reanalyzed our behavioral and imaging data by including a binary covariate for strategy in our behavioral and fMRI models (reported in Supplementary Tables S1–S3). Our results indicate that there were no significant modulatory effects of strategy use on the behavioral and imaging results of the economic game vignettes.

A total of four different vignette types were included in the experiment and varied along the experimental factors of Domain (describing the task type: life stories *vs* economic games) and Belief (identifying whether false belief were present or absent: false belief *vs* outcome description). Note that participants also performed half the trials under Threat induced through a probabilistic electric shock (threat present *vs* threat absent), which in the current analyses we control for, but do not specifically analyze (see Chang et al., manuscript in preparation, for this analysis). Specifically, in the Life Story-Outcome condition, the participants were reading about events that happen to another person. They were asked to answer questions about an objective description of the consequence of the event. In the Life Story-Belief condition, the participants were explicitly asked about the most likely beliefs or intentions of the protagonist in the scenario. On the other hand, in the Economic Game-Outcome condition, the participants were asked to calculate the payoff of one of the interaction partners based on the rules of the economic game in question (TG or UG). Note that this condition not only served as a contrast condition in the economic game domain but also functioned as a manipulation check, allowing us to probe our participants’ understanding of the rules of the economic games reflected by (in)correct calculations of the payouts across different game contexts. Similar to the Belief condition in the life story domain, in the Economic Game-Belief condition, the participants were required to infer the (false) beliefs of the interaction partners during an economic game. Figure 1A shows example economic game vignettes (for the full list of economic game vignettes, see our project page on osf.io https://osf.io/3eg56/?view_only=face48878dd144848d26f1c7d3c47d31).

Finally, the vignettes based on the TG and UG were never presented together in the same block to avoid potential confusion and task switching. Robustness analyses on the performance accuracy and speed across these two game types are reported in the Supplementary Material. Furthermore, the different scenario types (i.e. life-belief, life-outcome, econ-belief and econ-outcome), game types (TG and UG) and scenario topics (e.g. computer error scenarios) were pseudo-randomly distributed across Threat conditions.

#### Task description

Figure 1B illustrates the sequence and timing of a representative block and trial. Each block started with a block cue informing participants of the condition throughout the current block (3000 ms). Conditions varied based on the factors of Domain (life story *vs* economic games), Belief (false belief *vs* outcome description) and Threat (threat present *vs* threat absent) and were randomized throughout the experiment and for each participant. The example in Figure 1B shows an Economic Game-Belief-NoThreat condition, indicating that the three vignettes in the current block contain economic game scenarios, in which participants were asked to infer the interaction partner’s intentions and beliefs, and they did not receive electric shocks throughout this block. The block cue was then followed by a blank screen containing a fixation cross for a jittered duration (range: 3500–4750 ms, mean: 4000 ms). Thereafter, participants were asked to read the current vignette, for which they were given 10 000 ms. This period is referred to as the ‘vignette period’ below, during which participants read about a sequence of events that enabled them to develop an understanding of the protagonist’s beliefs in the Belief condition as illustrated in Figure 1A. The vignette display was followed by a ‘question period’, which was self-paced and terminated after 7000 ms. During this period, participants were required to integrate the information gathered during the vignette period to answer incentivized questions about the beliefs of one of the protagonists in the Belief condition, as illustrated in Figure 1A. Participants chose from two possible options, one incorrect and one correct one, with the position of the correct option randomized across trials. The correct answers were incentivized at a piece rate of 0.2 Euros to ensure that participants maintain attention and motivation throughout the experiment (Contreras-Huerta *et al.*, 2020). It was therefore in the best interest of participants to answer correctly and within the 7000 ms period, as otherwise they would forgo payment for that trial. Feedback was shown for 500 ms as soon as the participants pressed the corresponding button of the option, or after the 7000 ms period expired with no button press. Feedback indicated whether responses were correct, incorrect or too slow. Note that participants were not able to move through the experiment faster by responding faster during the question period as the remainder of the question period was added to the feedback duration if RT < 7000 ms. An additional jitter period (range: 25 000–7000 ms, mean: 4000 ms) was added at the end of each trial before the next trial started. Given our use of a hybrid design, a rest period of 11 000 ms was added at the end of each block to allow the blood-oxygenation-level-dependent (BOLD) signal to return to baseline. Each participant completed a total of 96 trials distributed across 32 blocks and 4 runs. The task was programmed and presented in MATLAB 2017b using the Cogent toolbox (http://www.vislab.ucl.ac.uk/cogent.php). Task stimuli were projected on a screen at the scanner head and were visible to the participants via a mirror mounted onto the head coil.

#### Payment determination

Participants earned a €0.20 bonus for each correct answer that was provided within the time limit of 7 s. The final payment for participation consisted of the performance bonus (max. €19.20) and the endowment of €14 paid for completing the online survey before the fMRI experiment. Participants earned an average of €32.32.

#### fMRI data acquisition

The fMRI data were collected using a 3.0 Tesla Philips Achieva scanner located at the Behavioral Science Lab at the University of Amsterdam. T1-weighted structural images were acquired axially in ascending slice order with the following parameters: 1 × 1 × 1 mm^3^ voxel size resolution with 220 slices, TR = 8.2 ms, TE = 3.7 ms, flip angle = 8°). Functional images were acquired axially in ascending slice order using a T2*-weighted gradient-echo, echo-planar pulse sequence with the following parameters: 3.0-mm slice thickness, 3.0 × 3.0 mm^2^ in-plane resolution of 36 slices with a slice gap = 0.3 mm, TR = 2000, flip angle = 76.1° and with 240x240-mm field of view). In addition, to correct echo planar images (EPI)s for signal distortion, we also conducted an additional field map scan at the half-way point of the experiment using a phase difference (B0) scan with slices acquired axially in ascedning slice order and the following parameters: 2.0 × 2.0 × 2.0 mm^3^ voxel size resolution without slice gap, TR = 11 ms, TE_s_ = 3 ms, TE_l_ = 8 ms, flip angle = 8°).

#### fMRI preprocessing and analyses

Imaging data analysis was carried out with SPM12 (Wellcome Department of Cognitive Neurology, London, UK) and the CONN toolbox (Whitfield-Gabrieli and Nieto-Castanon, 2012). Preprocessing followed the following steps: First, all functional images were simultaneously realigned to the first volume of the first run using septic b-spline interpolation and unwarped (using B0 maps) using the realign and unwarp function in SPM12, followed by slice timing correction. Afterward, T1-weighted structural images were co-registered with the functional images and then segmented into six different tissue classes using the segment function in SPM12. Next, all images were normalized to the Montreal Neurological Institute T1 using the forward deformation parameters from segmentation. Finally, all functional images were smoothed using spatial convolution with a Gaussian kernel of 6 mm at full-width half maximum (FWHM).

Statistical analyses were carried out using the general linear model. To reflect our factorial design, the model included separate regressors of interest for each Domain (life story *vs* economic games) and Belief (false belief *vs* outcome description) condition. These regressors were modeled separately for the vignette and question periods. Our model, therefore, included a total of eight regressors of interest: (1) false belief and (2) outcome vignettes in the context of life stories, and (3) false belief and (4) outcome vignettes in the context of economic games, which were modeled during both the vignette and question periods. Regressors of interest were modeled using a canonical hemodynamic response function. To best capture mentalizing during the question period, we used a variable epoch model from the onset of the question until option choice (button press). We also modeled regressors of no interest, which include each block cue, the feedback period, shock moment and the Threat condition (threat present *vs* threat absent), as well as omitted trials in which no response was provided by the participants. While omissions were rare (on average 0.55%), these were modeled explicitly to ensure that we only included trials for which we are certain that participants paid attention to the task. In addition, the six motion parameters derived from the realignment procedure were modeled as regressors of no interest. All results were Family-Wise Error (FWE)-corrected at the cluster level with a cluster-forming threshold of *P* < 0.001.

Conjunction analyses were conducted to test the overlap between belief-based activations in the life story and the economic game domains and were based on the conjunction null (Nichols *et al.*, 2005). Whole-brain statistical maps for each domain used a voxel threshold at an alpha value of *P* < 0.001 and were FWE-corrected at the cluster level. (For completeness, we also report the uncorrected results in Table 5.) The individual maps were then multiplied together using the ImCalc function in SPM12, which creates a map of voxels that are significantly activated in both conditions, reflecting a logical ‘and’ conjunction (Nichols *et al.*, 2005).

#### Connectivity analyses

Generalized Psychophysiological Interaction (gPPI) analyses were conducted using the CONN functional connectivity toolbox (www.nitrc.org/projects/conn) (Whitfield-Gabrieli and Nieto-Castanon, 2012) using two analyses: (1) region of interested (ROI)-to-ROI analysis to identify the specific interconnectivity among a restricted set of regions of interest that are commonly associated with social cognitive processes and (2) seed-based, whole-brain (seed-to-voxel) analysis to identify the wider connectivity of these social cognition regions with additional brain areas. The data were first prepared for connectivity analyses by preprocessing the fMRI data using the indirect segmentation and normalization pipeline in CONN, which is largely equivalent to our preprocessing steps described earlier, but included an additional step of identifying and removing outlier scans from the analysis (Artifact Detection Tools). Next, the data underwent denoising. In accordance with the anatomical component-based noise correction method (aCompCor, Behzadi *et al.*, 2007; Muschelli *et al.*, 2014), denoising was conducted before functional connectivity analyses and included 10 cerebrospinal fluid (CSF) and 10 white matter principal components as nuisance covariates, as well as 6 realignment parameters, their first-order temporal derivatives and quadratic effects (24 parameters in total), the outlier scans identified by Artifact Detection Tools and all task effects and their first-order derivatives (48 parameters in total). Low-frequency fluctuations were isolated using a low-pass temporal filter (0.008 Hz) after denoising. Thresholding for ROI-to-ROI analyses was done using the Threshold-free cluster enhancement (TFCE) method (Smith and Nichols, 2009) using the CONN default setting (hmin=1, E=0.5, and H=2) and peak-level family-wise error corrected *P*-values.

**Fig. 4.**
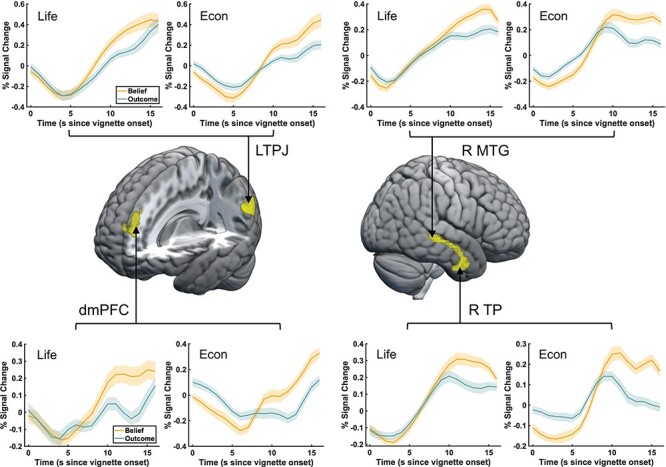
Conjunction analysis during the vignette period. A conjunction analysis showed significant overlap between the economic games and standard FBTs in a wider network of social cognition regions, including the left TPJ, the dmPFC and the right middle temporal gyrus/temporal pole. Inlets show time courses of significant activations plotted separately for the life story and economic game domains. Time courses were extracted from voxels in the regions identified by the conjunction analysis, which were further thresholded to separate clusters in the middle temporal gyrus. The shaded area denotes the standard error of the percentage signal change.

Seed regions for functional connectivity analyses were extracted from the conjunction maps assessing the overlap of belief-based activation (assessed via the contrast belief *vs* outcome) during the economic game and story-based task domains for vignettes (see Figure 4) and question periods (see Figure 6). Note that because we focus on regions that were jointly activated during the economic games and standard FBT and therefore have similar belief-based activation profiles, we did not distinguish between these task domains in connectivity analyses and analyzed belief-based connectivity (belief  > outcome) independent of Domain. Furthermore, to ensure that current activations match social cognition regions from prior studies, these conjunction maps were further conjoined with the smoothed (FWHM kernel of 1 mm) neurosynth map obtained via an association test for the meta-analysis term ‘mentalizing’. To remove smaller regions, we used a cluster threshold of *k* ≥ 25, which led to the following seed regions (maps with our seed regions can be found on our project page on osf.io): (i) during the vignette period, seed regions for connectivity analyses included the dmPFC (6, 56, 23, *k* = 46), the left TPJ (−48, −58, 26, *k* = 89), the right TP (48, 2, −31, *k* = 79) and the right medial temporal gyrus (MTG) (51, −28, −4, k = 26); (ii) during the question period, seed regions for connectivity analyses included the left TPJ (−60, −61, 20, *k* = 99), the dmPFC (−6, 56, 26, *k* = 55), the left MTG (−54, −28, −4, *k* = 112) and the bilateral TP (left: −54, 5, −25, *k* = 168, right: 48, −4, −37, *k* = 178).

**Fig. 5.**
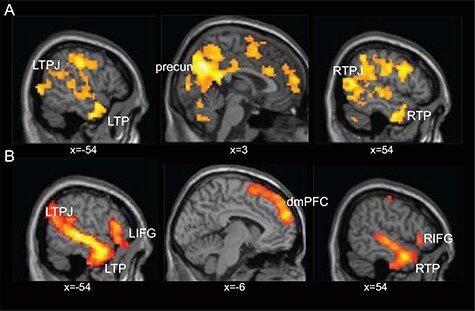
Whole-brain analysis of belief activations during the question period for the contrast belief  > outcome in the life story domain (A) and economic game domain (B). The results show consistent activations in mentalizing regions in both tasks, particularly in the dmPFC, the bilateral TPJ and the temporal pole. The results shown here were FWE-corrected at the cluster level with a cluster-forming threshold of *P <* 0.001.

**Fig. 6.**
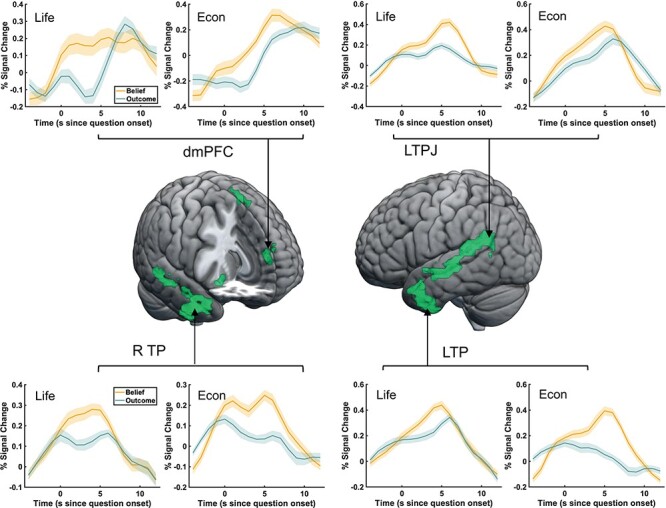
The conjunction analysis during the question period. A conjunction analysis showed significant overlap in a wider network of mentalizing regions, including the left TPJ, the dmPFC and the bilateral temporal pole. Inlets show time courses of significant activations plotted separately for the life story and economic game domains. Time courses were extracted from voxels in the regions identified by the conjunction analysis, which were further thresholded to separate clusters in the middle temporal gyrus. The shaded area denotes the standard error of the percentage signal change.

#### Behavioral results

The focus of our behavioral analyses was to test whether our novel economic game vignettes yielded behavior that is comparable to the standard life story vignettes in terms of overall accuracy and reaction times. At first glance, there seem to be only small differences in accuracy and reaction times across the two domains, with average accuracy reaching 95% for both the life story domain and the economic game domain. Closer inspection, however, revealed differences between the domains that seem to be largely driven by differences between the Outcome conditions in the life story and economic game vignettes (see Figure 2). This difference is likely due to the Economic Game-Outcome condition requiring computations of payouts, whereas standard vignette outcome trials only required an understanding of the story line. In contrast, for both the Economic Game and the Life Story-Belief conditions, participants had to understand the intentions and beliefs of the protagonist.

To analyze the behavioral data, we conducted logistic regressions implemented in the context of a generalized linear mixed-effects model. Models included responses on each trial (correct/incorrect) and log reaction time as dependent variables, as well as the Domain and Belief conditions as fixed-effects predictor variables and Threat as a fixed-effects control variable. Models were estimated via the mixed function of the AFEX package in R (Singmann *et al.*, 2016) that relies on the lme4 package. We report results from models with the maximum possible random-effects structure (Barr, 2013). For reaction times, linear regressions using a full model structure with random slopes for the Domain and Belief factors, in addition to random intercepts were employed. For accuracy, logistic regressions were used. Including all random slopes led to overfitting, requiring us to reduce the number of random slopes, such that all final models include a subject-wise random intercept, and a subset includes a random slope for the Domain factor. Note further that we report analyses for the pilot experiment, the fMRI experiment and the combined dataset in all tables but focus our discussion of the results on the data collected during the fMRI experiment. Please see the supplementary materials for the equations describing the winning models.

#### Accuracy across the Belief and Domain conditions

As reflected in Figure 2A, we find a significant main effect of Belief (*X*^2^ = 16.83, *P* < 0.001) on accuracy and a significant interaction between Belief and Domain (*X*^2^ = 17.85, *P* < 0.001). Follow-up tests of the interaction were conducted using the free method implemented via the multcomp package (Hothorn *et al.*, 2008). The results from pairwise comparisons using the Sidak correction indicate that these effects are due to a significantly lower accuracy in the economic games compared to the life story task in the Outcome condition (estimate = −0.82, *Z* = −2.60, *P* = 0.018), while only a near-significant difference between the economic games and life stimuli was observed in the Belief condition (estimate = 0.75, *Z* = 1.91, *P* = 0.056).

This result indicates that accuracy differences were only found in the Outcome, but not in the Belief condition of the Belief Factor. The Economic Game-Outcome condition has different cognitive demands compared to those of all other conditions as it requires computations of payouts, which is reflected by the current results. Note that, except for the belief main effect, these results do not replicate across different datasets and model specifications (Table 1). Moreover, while the actual effects fall in the range between 1.3% and 4.7% and are therefore relatively small, they do reach significance and are driven by our economic games stimuli. Finally, two robustness analyses indicate that the accuracy results are not qualified by different strategies used by participants (see SM Robustness Analysis 1, Supplementary Table S1 and also see Supplementary Table S3 for fMRI robustness check), but that results in the Outcome condition are significantly affected by the different games used for the economic games vignettes (see SM Robustness Analysis 2, Supplementary Table S4).

#### Reaction time across the Belief and Domain conditions

Figure 2B shows the mean reaction times across the Domain and Belief conditions. We analyzed the log reaction times of the correct trials only and found significant main effects of Belief (*Χ*^2^(1) = 4.52, *P* = 0.033) and Domain (*Χ*^2^(1) = 63.99, *P* < 0.001) and a significant interaction between Belief and Domain (*Χ*^2^(1) = 69.51, *P* < 0.001, see Table 2 for ANOVA tables from mixed models). Follow-up pairwise tests of the interaction were conducted using the free method from the multcomp package via the Sidak correction. The results indicate a significant difference between economic games and life stimuli in the Outcome condition (estimate = −0.469, *t* = −18.81, *P* < 0.001), while no significant difference between the economic games and life stimuli was observed in the Belief condition (estimate = −0.046, *t* = −1.88, *P* = 0.064). These results indicate that in the Outcome condition, response times were significantly faster for economic games, while participants spend about equally long answering questions about beliefs in the economic games and standard FBT. This again agrees with the deviation of behavior with this type of stimulus from the other vignette stimuli. Note that our fMRI models implicitly control for these reaction time differences by implementing a variable epoch model for all question period regressors (Grinband et al., 2008). Similar to accuracy, the robustness analyses indicate that the reaction time results are not qualified by different strategies used by participants (see SM Robustness Analyses 1: Supplementary Table S2), but that results in the Outcome condition are significantly affected by the different games used in the economic games vignettes (see SM Robustness Analyses 2, Supplementary Table S4). In an additional analysis (see Robustness Analyses 3, Supplementary Figure S4, Supplementary Table S5), we also test for speed accuracy trade-offs in each of our experimental conditions. We do not find speed accuracy trade-offs in the economic game scenarios, and correcting for speed accuracy trade-offs using the Balanced Integration Score (Liesefeld and Janczyk, 2019) does not change results.

### fMRI results

#### Mentalizing effects during belief formation in the vignette period across DRomains

In our initial analyses, we focus on the vignette period during which participants were required to form a belief about the protagonists’ mental state by reading about a sequence of events. To test whether our economic game vignettes elicit similar activation patterns in social cognition regions as standard FBT vignettes, we first identify the neural correlates of mentalizing via the contrast belief  > outcome and did this separately for economic and life story vignettes. For the life story vignettes, our results replicate previous findings (Saxe and Kanwisher, 2003; Van Overwalle, 2009; Bruneau *et al.*, 2012; Schurz *et al.*, 2014), as we find significant activation in the bilateral temporal parietal junction (the left TPJ: −51, −55, 29, *k* = 678; the right TPJ: 54, −49, 23, *k* = 1094), the dmPFC (0, 47, 32, *k* = 427), the precuneus (3, −58, 38, *k* = 145) and also the bilateral inferior frontal gyrus (IFG; the left IFG: −30, 20, −19, *k* = 72; the right IFG: 57, 26, −10, *k* = 140) (Figure 3A, Table 3). For the novel economic game vignettes, we find a less distributed set of social cognition regions that include the dmPFC (−9, −53, 29, *k* = 246), the left TPJ (−54, −70, 32, *k* = 106), the right TP (51, −10, −37, *k* = 310) and the left temporal gyrus/TP (−48, −1, −25, *k* = 276) (Figure 3B, Table 3).

**Table 3.** Activations from whole-brain analyses of the mentalizing effect during the vignette period in the life story and economic game domains (*P* < 0.05 FWE-corrected at the cluster level)

Structure		L/R	Cluster Size	*x*	*y*	*z*	Peak *t*
	Belief > Outcome (Life Story)						
TPJ		R	1094	54	−49	23	7.45
TPJ/supramarginal gyrus		L	678	−51	−55	29	6.99
dmPFC		Bil.	427	0	47	32	5.93
Inferior frontal gyrus		L	72	−30	20	−19	5.33
Precuneus		Bil.	145	3	−58	38	5.29
Inferior frontal gyrus		R	140	57	26	−10	5.11
Frontal eye fields		R	73	−48	20	44	4.78
Frontal lobe		L	79	−57	35	−4	4.49
	Belief > Outcome (Economic Game)						
Middle temporal gyrus/temporal pole		L	276	−48	−1	−25	6.26
dmPFC/superior frontal gyrus		L	246	−9	53	29	5.84
Temporal pole		R	310	51	−10	−37	5.78
TPJ/angular gyrus		L	106	−54	−70	32	5.05

To test the overlap of these two networks, we performed a conjunction analysis of the FWE-corrected maps shown in Figure 3 reflecting belief activations (belief *vs* outcome) in the economic game and story-based task domains. Thereby, we examine which voxels showed significant belief-based activation across both versions of the FBT, i.e. the life story and economic games domain. The conjunction analysis identified significant overlap in social cognition regions for both domains, specifically in the left TPJ (−51, −61, 26, *k* = 91), the dmPFC (−6, 47, 35, *k* = 46) and the right temporal gyrus (48, −25, −4, *k* = 158) (see Figure 4 and Table 4, top part). Moreover, we extracted activation patterns from regions that showed significant activation in both the Life Story and Economic Game conditions and plot their time course. Inlets in Figure 4 illustrate that, in both the life story and economic game vignettes, in accordance with the relatively sustained nature of this task phase activity in these regions rises after about 5 s and, importantly, show higher peak values in the Belief condition compared to the Outcome condition. These results support the notion that this network of regions is involved in mentalizing in both domains, namely life stories and economic games.

**Table 4.** Results from conjunction analyses for vignette and question periods. The regions activated in both the economic game and life story domains were identified by conjoining the two statistical maps, which were each thresholded via a cluster-forming *P* value of <0.001 and an FWE-corrected cluster threshold. Additional regions are listed in italics that reflect a less conservative conjunction analysis based only on a cluster-forming threshold of *P* < 0.001 but without FWE-corrtion

Structure		L/R	Cluster size	*x*	*y*	*z*
	Conjunction of mentalizing effect during the vignette period					
Superior temporal gyrus		R	158	48	−25	−4
dmPFC		Bil.	46	−6	47	35
TPJ		L	91	−51	−61	26
*TPJ*		*R*	*18*	*48*	*−55*	*23*
*Middle temporal gyrus*		*L*	*11*	*−60*	*−10*	*−13*
*Temporal pole*		*L*	*10*	*−54*	*−7*	*−31*
*Precuneus*		*Bil.*	*7*	*−6*	*−55*	*29*
	Conjunction of mentalizing effect during the question period					
Temporal pole		L	203	−54	5	−25
Temporal pole		R	434	48	−7	−37
Middle temporal gyrus/TPJ		L	455	−54	−28	−4
Cerebellum		R	39	24	−73	−37
Putamen		R	43	24	17	−7
dmPFC		Bil.	55	−6	56	26
SMA/pre-SMA		Bil.	44	−3	8	65
*Cerebellum*		*L*	*25*	*−27*	*−76*	*−40*
*Precuneus*		*Bil*	*18*	*−3*	*−55*	*29*
*Temporal pole*		*L*	*5*	*−27*	*8*	*−31*
*Pre-motor area*		*L*	*5*	*−48*	*−4*	*50*

**Table 5.** Activations from whole-brain analysis of the mentalizing effect during the question period in each domain (*P* < 0.05 FWE-corrected at the cluster level). The regions listed in italics are subclusters within larger activation clusters. The subclusters were further identified using small volume correction (svc) for each TPJ cluster from the neurosynth map obtained via an association test for the term ‘mentalizing’. All listed regions are based on FWE-corrected whole brain analyses, except for regions listed in italics, which are small volume corrected (svc)

Structure			L/R	Cluster size	*x*	*y*	*z*	Peak *t*
		Belief > Outcome (Life Story)						
Precuneus (extending into)			Bil.	13923	−3	−67	32	7.44
	*TPJ (svc)*		*L*	*22*	*−57*	*−58*	*23*	*4.33*
	*TPJ (svc)*		*R*	*146*	*48*	*−67*	*14*	*6.1*
Temporal pole			L	219	−54	−4	−34	5.75
d orsolateralPrefrontalCortex (DLPFC			L	524	−24	44	35	5.39
		Belief > Outcome (Economic Games)						
Superior temporal gyrus (extending into)				2698	−57	−28	−1	12.67
	*TPJ (svc)*		*L*	*164*	*−63*	*−61*	*20*	*7.41*
Temporal pole			R	976	45	8	−28	8.77
Medial PFC			Bil.	831	−9	59	32	8.63
Precentral gyrus			R	112	66	−4	29	5.72
Posterior insula			R	119	39	−16	17	5.70
Sensorimotor cortex			R	131	45	−25	65	5.70
Posterior cerebellum			R	76	24	−73	−37	5.68
Inferior frontal regions			R	83	51	26	2	5.30
Putamen			R	120	24	11	−7	4.91

#### Mentalizing effects during belief inferences in the question period across Domains

Next, we investigated the period during which participants answered questions concerning the events described in the vignettes. This period required participants to make inferences about the understanding they formed about the protagonists’ beliefs and intentions from the sequence of events described in the life stories and economic interactions to correctly answer the incentivized questions. Since this period required an integration of the information gathered during the vignette period with what was asked in the question, we expected more extended activation patterns that primarily include social cognition regions during this period. We again contrasted Belief *vs* Outcome conditions to test the effect of mentalizing and did so separately for the economic game and life story vignettes. In the life story domain, shown in Figure 5A and Table 5, we identified three large clusters with peaks in the precuneus (−3, −67, 32, *k *= 13 923), the left TP (extending into the TPJ; −54, −4, −34, *k* = 219) and the left dorsolateral Prefrontal Cortex (dlPFC) (extending into the dmPFC; −24, 44, 35, *k* = 524). For questions concerning economic games, shown in Figure 5B and Table 5, we identified a network that includes bilateral temporal gyrus, with the left region extending into the TPJ (−57, −28, −1, *k* = 2698), the right TP (45, 8, −28, *k* = 976), the dmPFC (−9, 59, 32, *k* = 831), the right sensorimotor cortex (45, −25, 65, *k* = 131), the right posterior cerebellum (24, −73, −37, *k* = 76), the right IFG (51, 26, 2, k = 83), the right insula (39, *y* −16, 17, *k* = 119) and right putamen (24, 11, −7, *k* = 120).

Next, similar to the approach for the vignette reading period, we examined the overlap of the networks recruited in both the life story and economic game domains via a conjunction analysis of the FWE-corrected maps shown in Figure 5, reflecting belief activations (belief *vs* outcome) in the economic game and story-based task domains. The conjunction results are shown in Figure 6 (see also Table 4, bottom part) and confirm that significant belief-based activation occurred in a network of overlapping regions in the life story and economic game domains. The areas that are activated across these conditions include the dmPFC (−6, 56, 26, *k* = 55), the left middle temporal gyrus extending into the TPJ (−54, −28, −4, *k *= 455), the left TP (−54, 5, −25, *k* = 203), the supplementary motor cortex (−3, 8, 65, *k* = 44), the right temporal gyrus extending into a TP (48, −7, −37, *k* = 434) and the right posterior cerebellum (24, −73, −37, *k* = 39).

Moreover, we extracted activation patterns from regions that showed significant activation in both the Life Story and Economic Game conditions and plotted the respective time courses. Inlets in Figure 6 illustrate that, in accordance with the more transient nature of this task phase, activity in these regions rises almost immediately after the onset of the question period and peaks at about 6 s. Time courses also show a larger peak in the Belief condition compared to the Outcome condition for both the life story and economic game vignettes. These results support the notion that this network of regions is involved in mentalizing in both the life stories and economic game domains during the question period.

#### Functional connectivity during mentalizing

In our final analyses, we asked the question to what extent the regions identified by the conjunction analyses between our economic game and story-based vignettes are functionally interconnected with other social cognition regions during mentalizing. To this end, we conducted gPPI analyses. First, using ROI-to-ROI analyses, we inspected the belief-based (belief *vs* outcome) interconnectivity within our set of ROIs during each of the task phases. Next, using seed-based whole-brain analyses, we assessed whether additional target regions showed stronger positive connectivity with our seed regions during the Belief condition relative to the Outcome condition. Analyses were conducted separately for the vignette and question periods, and for each ROI-to-ROI and seed-based analysis, we used as seeds those regions that were identified by the conjunction analysis for that specific period (see ‘Materials and Methods’ section).

**Fig. 7.**
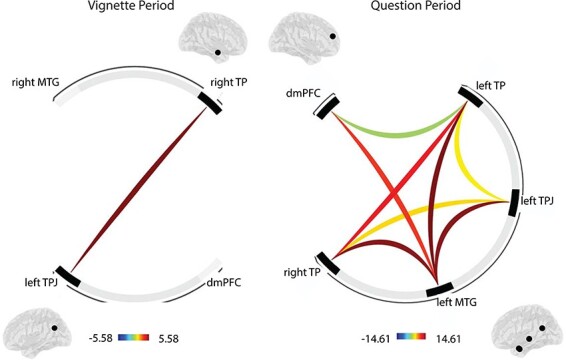
Functional connectivity among ROIs during the vignette and question periods. ROI-to-ROI analyses show heightened connectivity during the Belief condition relative to the Outcome condition between the left TPJ and the right TP during the vignette period (left ROI-ring display, TFCE value = 5.58, FWE-corrected *P *= 0.044) and extensive interconnectivity among ROIs during the question period (right ROI-ring display, TFCE value = 14.91, FWE-corrected *P* = 0.009).

During the vignette period, we find that the left TPJ shows significant interconnectivity with the right TP (Figure 7 left panel, TFCE value = 5.58, FWE-corrected *P* = 0.044), indicating relatively restricted interconnectivity within our network of ROIs. This could be due to a mismatch between the sustained nature of the vignette period and how regions in fact communicate throughout this period, such that the fluctuation of transient and repeated communication between regions might not be picked up by the current regression analysis. In the shorter question period, we see extensive interconnectivity between all the social cognition regions we included as ROIs (Figure 7, right panel, TFCE value = 14.91, FWE-corrected *P* = 0.009). This indicates that preparing an answer that involves an understanding of beliefs requires strong cross-talk between social cognition regions.

**Fig. 8.**
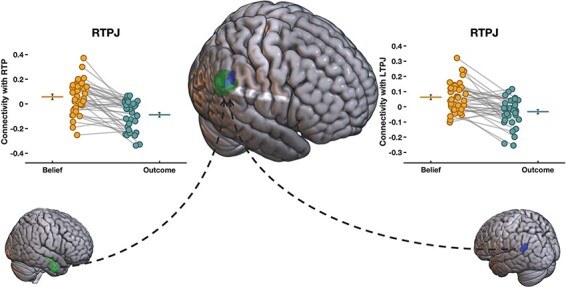
The whole-brain gPPI analysis of belief-based effective connectivity during the vignette period. The gPPI analyses show heightened connectivity during the Belief condition relative to the Outcome condition between the left TPJ seed and the right TPJ target during the vignette period (58, −52, 30, *k* = 126, cluster-level FWE-corrected *P* = 0.0253) as well as between the right TP seed and the right TPJ target (52, −54, 26, *k* = 570, cluster-level FWE-corrected *P* < 0.0001).

For the whole-brain gPPI analyses in this study, we observe an interesting pattern that highlights the role of the right TPJ during the vignette period, which is a target region of both the left TPJ (left to right TPJ: 58, −52, 30, *k* = 126, cluster-level FWE-corrected *P* = 0.0253) and the right TP (the right TP to the right TPJ: 52, −54, 26, *k* = 570, cluster-level FWE-corrected *P* < 0.0001) during belief relative to outcome vignettes (Figure 8). This result is interesting, as it confirms the role of the right TPJ in mentalizing, which we do not find in conjunction with analyses reported earlier, and shows the importance of a wider interconnected set of regions involved in mentalizing during the vignette period. We also find reduced connectivity between the TP seed region and its target in the sensorimotor area (−26, −30, 66, *k* = 177, cluster-level FWE-corrected *P* = 0.0038).

During the question period, we find enhanced belief-based connectivity between the left TPJ and its target in the right cerebellum (Supplementary Figure S2; 24, −78, −18, *k* = 169, cluster-level FWE-corrected *P* =0.0059). Finally, the dmPFC shows enhanced belief-based connectivity with a region in the superior parietal lobe that extends to the precuneus (Supplementary Figure S3; −24, −66, 48, *k* = 141, cluster-level FWE-corrected *P* = 0.0150).

## Discussion

An important question in the field of social neuroeconomics is whether the activations within brain regions that are meta-analytically associated with mentalizing and that are also consistently involved in decisions in the context of interactive economic games (e.g. Fehr and Camerer, 2007; Rilling & Sanfey, 2011; Alós-Ferrer and Farolfi, 2019; Engelmann *et al.*, 2019) indeed reflect mentalizing about interaction partners. While this conjecture is theoretically plausible and is supported by the stark overlap of activation patterns across a variety of tasks that are associated with belief inferences (Van Overwalle, 2009; Mar, 2011; Schurz *et al.*, 2014; Molenberghs *et al.*, 2016), it is important to compare and identify the overlap between the neural systems engaged in mentalizing across different contexts, including not only in life events but also in an economic games context, in the same participants using the same task. The goal of the current study was to address this gap in the literature using a novel version of the FBT that required our participants to make belief-based inferences in the context of economic game scenarios.

The fMRI results in our study indeed identify a strong overlap between the networks engaged during the standard FBT and a modified version that requires an understanding of economic games to correctly infer the beliefs of interaction partners in hypothetical economic games. This shows that our novel economic games FBT, which asked participants to observe two agents interact in the TG and UG and make inferences about their beliefs, reliably activating canonical social cognition regions. Specifically, using conjunction analyses, we find two regions that show enhanced activity during belief-based (relative to outcome-based) inferences during both variants of the task, namely the left TPJ and the dmPFC. This finding is in line with a series of previous meta-analyses on the neural underpinnings of mentalizing, which consistently pinpointed these two nodes as core areas for mentalizing across different paradigms, including economic games (Van Overwalle, 2009; Mar, 2011; Schurz *et al.*, 2014; Molenberghs *et al.*, 2016). Moreover, we find that these regions are involved in reasoning about others’ beliefs during two periods of our task: the vignette period, during which participants need to read and understand the beliefs of others, and the question period, which required them to integrate the information gathered via the vignettes and answer a brief question about the protagonists’ beliefs. The consistency of the activation overlap across the different task types and task periods further underlines the importance of these regions for belief-based inferences. Moreover, these results implicate the left TPJ and the dmPFC in belief-based inferences in the context of economic games. Interestingly, the location of the TPJ activation, due to a conjunction between activations in the economic games and standard FBT, in the left hemisphere is consistent with a recent observation of the left TPJ during trust decisions (Engelmann *et al.*, 2019), as well as a recent meta-analysis of the neural correlates of TPP (Bellucci *et al.*, 2020). Jointly, the results in our study substantiate the notion that the commonly observed activation of social cognition regions during interactive economic games, particularly the left TPJ and the dmPFC, reflects mentalizing (Fehr and Camerer, 2007; Rilling and Sanfey, 2011; Engelmann *et al.*, 2019).

The important role of the temporoparietal junction in mentalizing is further underlined by effective connectivity analyses. During the vignette period, the left TPJ shows enhanced belief-based connectivity with the right TPJ, and the right TPJ is a target of the right TP (Figure 8). This shows that even if the TPJ does not show bilateral activation in conjunction analyses, effective connectivity patterns implicate the bilateral TPJ during mentalizing in the vignette period. Moreover, connectivity patterns also underline the importance of cross-talk within a wider network of social cognition regions that include the bilateral TPJ, the bilateral TP and the dmPFC, when participants make belief-based inferences that involve mentalizing during the question period.

Our results, furthermore, indicate that there is a more extensive network of regions that are involved in belief-based inferences across the two task versions. This is clear from two types of analyses: (i) conjunction analyses of the overlap of activation patterns across standard and economic game FBT versions and (ii) effective connectivity analyses involving the regions identified in these conjunction analyses. The conjunction analyses identified more extended belief-based activation in the right TP (extending into the right middle temporal gyrus) during the vignette period and the bilateral TP during the question period. Moreover, the TP also showed heightened belief-based connectivity with target regions associated with social cognition, including the right TPJ during the vignette period (Figure 8) and the left TPJ and the left MTG during the question period. Our results of heightened belief-based activity and connectivity of the TP agree with its roles in semantic memory, face recognition and theory of mind (Gentileschi *et al.*, 2001; Gainotti *et al.*, 2003; Olson *et al.*, 2007), as all of these are social cognitive skills that support belief-based inferences (e.g. Patterson *et al.*, 2007). Moreover, this result is consistent with previous studies on the neural correlates of social cognitive (Frith and Frith, 2006) and social affective mechanisms (Völlm *et al.*, 2006).

As part of a more extended network of social cognition regions involved in mentalizing, the cerebellum deserves some additional discussion. Specifically, we find significant activation in the right posterior cerebellum during belief-based inferences in the question period (Table 4), and furthermore, the right posterior cerebellum is found as a target of the left TPJ in connectivity analyses (Supplementary Figure S2). Our results, therefore, substantiate the importance of the cerebellum as a region that supports mentalizing in important ways, but that falls outside of the typical social cognition areas within the cerebral cortex. In fact, a recent meta-analysis based on 350 fMRI studies provides strong support for the notion that the cerebellum subserves important social cognitive functions, particularly when a certain level of abstraction is required (Van Overwalle *et al.*, 2014). These social cognitive functions include mirroring others’ behavior, mentalizing and the representing abstract concepts in social contexts (e.g. group stereotypes). Our fMRI results support the hypothesis that the cerebellum is involved in belief-based inferences about others.

Moreover, the location of the cerebellum activation found in the current study corresponds well with what has been reported previously. Van Overwalle *et al.* (2014) suggest that right hemisphere lateralization of the cerebellum was specifically associated with mentalizing tasks that require language processing (Stoodley and Schmahmann, 2009), which matches the results reported here. Van Overwalle and Mariën (2016) examined the functional connectivity between the cerebellum and the cerebrum for mentalizing across five studies with a high level of abstractness (e.g. judgment of others’ traits and group stereotypes). They found significantly higher functional connectivity between the right posterior cerebellum and the bilateral TPJ and the dmPFC. Our results partially validate this prior finding, showing significantly higher belief-based functional connectivity between the left TPJ and the right posterior cerebellum during the question period. Taken together, our fMRI results are consistent with previous findings implicating the cerebellum in social cognitive processes and lend further support to the notion that the cerebellum is involved in belief-based inferences about others. It is therefore important for future studies in social neuroscience and social neuroeconomics to also examine the results in the cerebellum carefully.

### Limitations

As with every experiment, there are a number of limitations that need to be considered. The current paper presents a reanalysis of the data from a larger experiment on the effects of anxiety on the theory of mind. One of the limitations, therefore, is that participants completed the task in the context of threat blocks, in which they could experience electric shocks at unpredictable time points, and safe blocks, during which they were free from the threat of electric shocks. This approach is known to induce affective states of anxiety during threat blocks and relative safety during safe blocks (e.g. Engelmann *et al.*, 2015, 2019), and these affective states might enhance or depress the belief-based activation and connectivity of the regions reported in the current paper. We tackle this limitation by controlling for these effects and including the factor threat, as well as each electrical shock moment as regressors of no interest in all of our analyses. Given that these factors should mostly increase the noise in our data and work against our results, in conjunction with our activation and connectivity patterns being highly consistent with those previously reported in experiments and meta-analyses of the neural correlates of mentalizing (Van Overwalle, 2009; Mar, 2011; Schurz *et al.*, 2014; Molenberghs *et al.*, 2016), we are confident that our results are not an artifact of this manipulation.

A second limitation concerns our analyses of two separate periods of the task, the vignette period, during which participants were reading and forming an understanding of the events outlined in the vignette, and the question period, during which participants were asked to make inferences about what they just read. Our experimental design did not include jitter between these two periods, which would have allowed us to better separate the hemodynamic response across vignettes and question periods. We made this decision for three reasons: (i) to allow better comparison with previous studies (e.g. Saxe and Kanwisher, 2003; Young *et al.*, 2010a, 2010b), (ii) to ease the cognitive burden on our participants that jitter might have imposed, as suggested by the results from our behavioral pilot study and (iii) to keep the experiment relatively short. Moreover, this limitation is qualified by the BOLD patterns shown in Figures 4 and 6. We find during both task periods that BOLD responses follow the expected pattern, given the cognitive demands of that period. During the vignette period, BOLD responses rise to a peak between 10 and 15 s, reflecting the more sustained nature of social cognitive processes required to understand a sequence of events during this period. During the question period, we observe that the BOLD response starts from a low activation level (around zero percent signal change) and rises to a peak at around 5 s, reflecting the more transient nature of social cognitive processes during this period that is consistent with the average response time of 2.61 s during this period. Our findings that the BOLD responses during vignette and question periods follow patterns that are consistent with the cognitive demands of each period and that they start from a low activation level in the question period in regions that show overlap with those activated during the preceding period (the left TPJ and the dmPFC), therefore mitigate this concern.

Third, we need to point out that the control condition in the economic games FBT is different from the control condition in the standard FBT. While in the standard FBT, we used a story-based outcome condition, in the Economic Game-Outcome condition, our participants were asked to calculate the payoff of one of the interaction partners based on the rules of the economic game in question (TG or UG). While this leads to somewhat different behavioral results in this condition (Figure 2), we argue that the Economic Game-Outcome condition is nonetheless an ideal control condition for belief-based inferences made in the context of economic game vignettes. This is the case because participants need to apply the same understanding of economic games in both the Belief and Outcome conditions, but focus on different aspects of the social interaction, namely the interaction partners’ beliefs compared to their payouts (which are also a result of the social interaction). Furthermore, including the Economic Game-Outcome condition allowed us to ensure that participants understand the rules of the economic games and were able to calculate their payouts across different contexts.

Finally, our decision to study mentalizing in economic games from a third-party perspective has a number of advantages notably that mentalizing processes are not distorted by the affective and cognitive processes that support the decisions of participants directly involved in economic game interactions. However, this decision to use a third-person approach also comes with important trade-offs (Redcay and Schilbach, 2019). First, we do not investigate social decision-making processes *per se*, and the results therefore only speak to making inferences about players’ beliefs and intentions from the perspective of an outside observer. Moreover, interactions in economic exchange games are often sequential. In the example of the TG, the trustor decides on an initial transfer and the trustee chooses whether and how much to transfer back. Such sequential interactions may trigger very different social cognitive processes in first-party interactions compared to the third-party observation that was required from participants in the current study. There exist multiple theories about how first-person participants might approach TG interactions: (i) trustors send money because mutual trust maximizes utility (rational choice model), (ii) trusting behavior can be driven by injunctive norms (Dunning *et al.*, 2014), but (iii) can also be an expression of an expectation of reciprocity (Sapienza *et al.*, 2013), (iv) trusting might involve the assessment of a social risk of betrayal (Bohnet and Zeckhauser, 2004), (v) trust decisions might be boundedly rational and based on a reduced set of salient properties of the decision context (Evans and Krueger, 2016) and (vi) trust decisions might be based on a simulation of how one would behave in the role of the trustee (Engelmann *et al.*, 2019b). Our results showing social cognitive activations in observers of economic game interactions do not speak to the question of ‘how’ trust decisions are made but merely reflect that to understand and answer simple questions about false beliefs that arise in the context of economic game interactions relies on social cognitive processes that engage the left TPJ and the dmPFC. However, our results are also consistent with the notion that inferences about the false beliefs held by interaction partners in economic games are made by engaging mentalizing facilities. One possible explanation for our results that we favor is that this task is achieved by simulating how observers would act if they themselves were in the position of the interaction partners within the context outlined in the vignettes (see, for instance, Gallese and Goldman, 1998; Keysers and Gazzola, 2007; Waytz and Mitchell, 2011; Engelmann *et al.*, 2019b). As such, we posit that our results fill the gap between social neuroscience and social neuroeconomics by providing complementary evidence implicating activation within the social cognition network, but particularly in the left TPJ, in solving different false-belief contexts. However, there are important routes that the future studies could take to further complete the picture. For instance, the economic FBT task developed here could be useful as a localizer task in future fMRI studies interested in investigating mentalizing during decisions in economic games that require first-person interaction. One other promising direction of future research is a closer inspection of the decision strategies and beliefs of participants directly engaged in trust and back-transfer decisions to answer the important question of what drives decisions to trust or reciprocate.

### Conclusions

Our findings lend support to the notion that activations within the social cognition network that have consistently been observed during decisions in the context of interactive economic games reflect mentalizing about interaction partners. We addressed this question here by developing a novel version of the FBT that is based on interactions in economic games, specifically the TG and UG. Correctly answering questions about the beliefs of one of the players in the economic games FBT requires an understanding of the rules of these games. Comparing activation patterns during the standard story-based FBT with a novel game theoretic FBT in the same participants, we identify overlap between the neural systems engaged in mentalizing. Specifically, our conjunction analyses identify two regions that show enhanced activity during belief-based (relative to outcome-based) inferences during both variants of the task, namely the left TPJ and the dmPFC, which is in line with the results from previous meta-analyses (Van Overwalle, 2009; Mar, 2011; Schurz *et al.*, 2014; Molenberghs *et al.*, 2016; Bellucci *et al.*, 2020). Moreover, we find an extended network of regions that are important for mentalizing during both task versions, with the TP being prominently represented in conjunction and connectivity analyses, and the right TPJ showing enhanced connectivity with the left TPJ and the right TP during the vignette period. Jointly, our results support the notion that mentalizing during belief formation and inferences are supported by social cognitive processes in a wider network of social cognition regions that include the bilateral TPJ, the TP and the dmPFC as central nodes. Importantly, this is the case in the context of economic games and standard FBTs.

## Supplementary Material

nsad023_Supp

## Data Availability

The behavioral data and corresponding analysis scripts underlying this article are available on our project page on osf.io at the following link: https://osf.io/3eg56/?view_only=face48878dd144848d26f1c7d3c47d31.
