# Supplementary material for: Mentalizing in an economic games context is associated with enhanced activation and connectivity in the left temporoparietal junction
Source: Soc Cogn Affect Neurosci. 2023 May 2;18(1):nsad023. doi: 10.1093/scan/nsad023 (PMC10243902; doi:10.1093/scan/nsad023)
Supplement: nsad023_Supp [file nsad023_supp.zip › scan-22-074-File010.pdf]

Supplementary Materials

For

Mentalizing in an economic games context is associated with enhanced activation and connectivity in left temporoparietal junction

Li-Ang Chang[1], Konstantinos Armaos[2], Lotte Warns[3], Ava Q. Ma de Sousa[3,4,] Femke Paauwe[3], Christin Scholz[5], Jan B. Engelmann[1,6]*

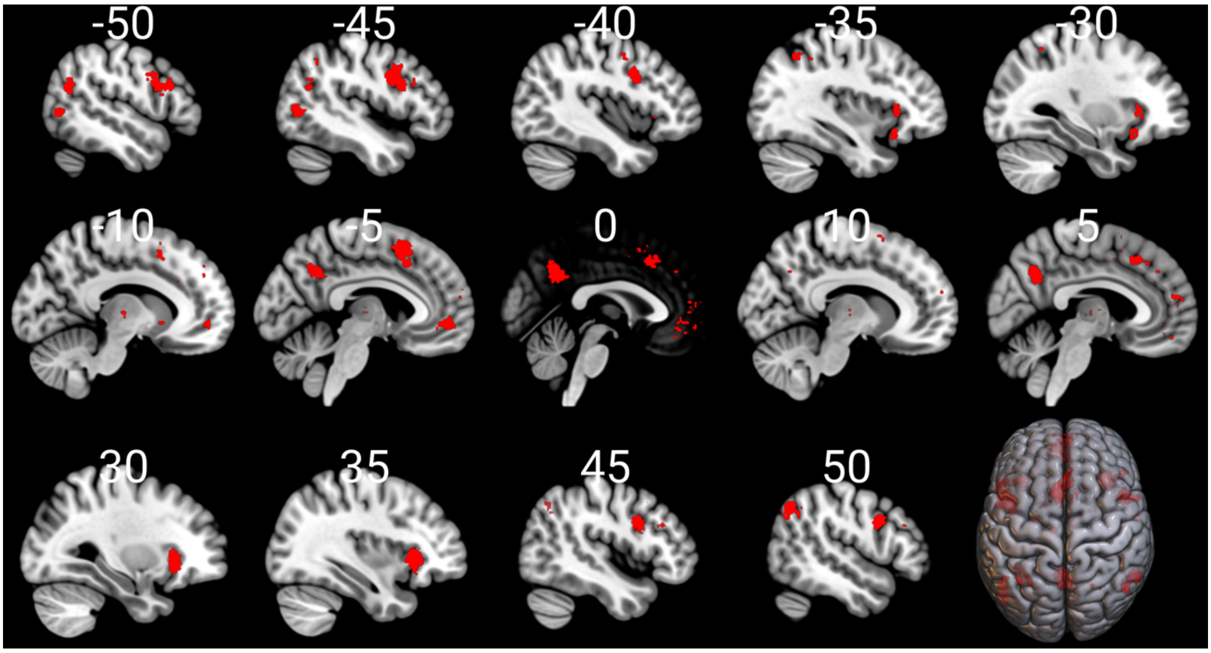

Figure S1. A conjunction analysis of two Neurosynth meta-analyses for the terms "game" (N=176) and "mentalizing" (N = 151) reveals overlap across these two tasks in canonical social cognition regions including bilateral TPJ, precuneus/PCC, and multiple clusters within dmPFC. Additional regions that show overlap include the vmPFC, AI and dlPFC. Note that uniformity tests were used for both meta-analyses to create this conjunction map due to a lack of results for the association test for "game". The conjunction was computed by multiplying the two neurosynth meta-analyses maps using the SPM tool imcalc.

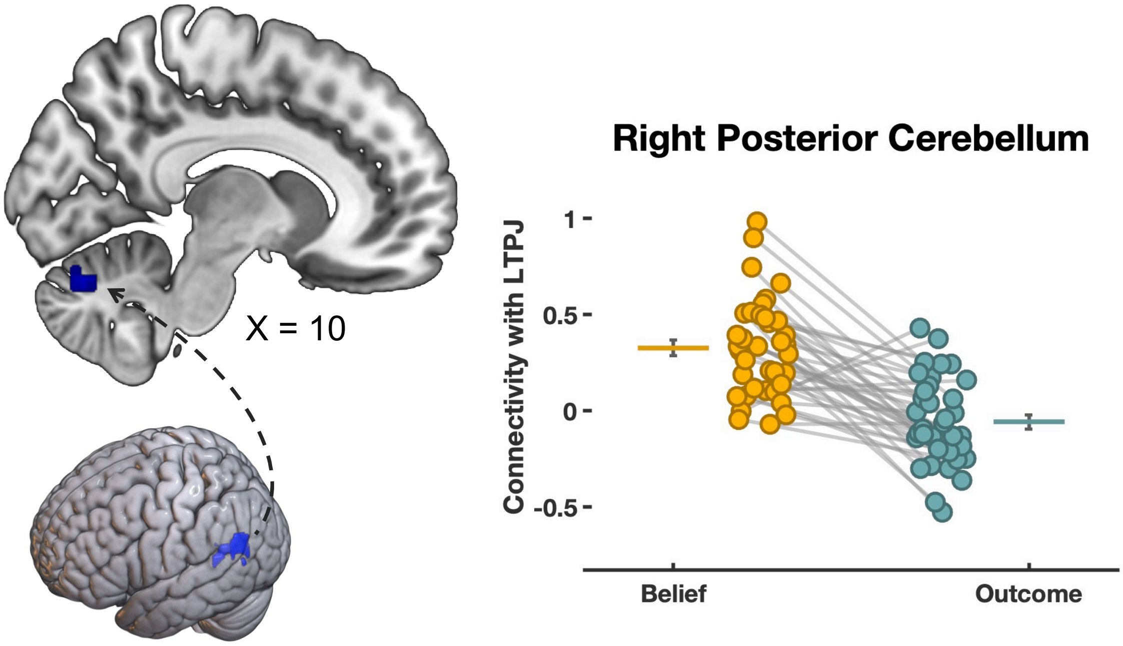

Figure S2. Question period whole-brain gPPI results showing the belief-based connectivity increases between the left TPJ seed and right posterior cerebellum target (24, -78, -18, k = 169, cluster-level FWE-corrected *p* =0.0059).

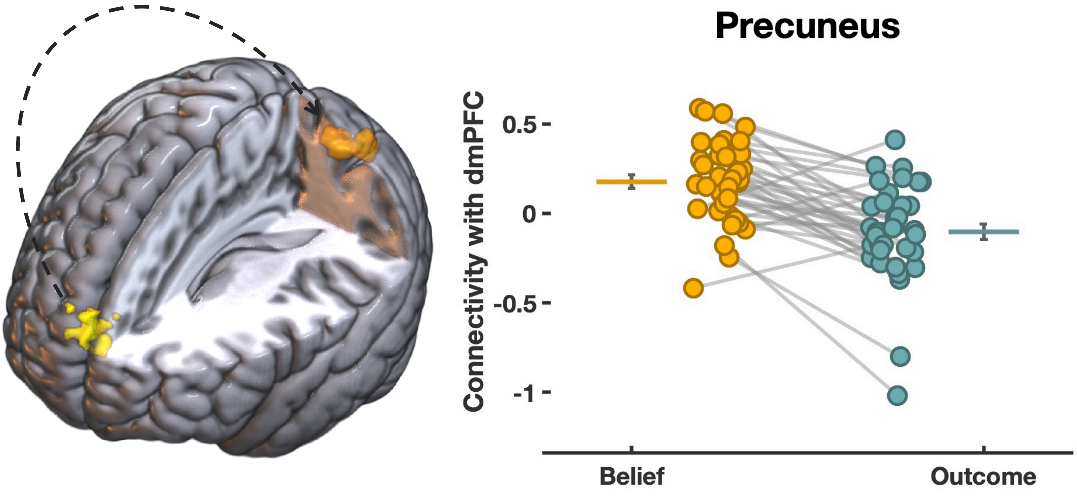

Figure S3. Question period whole-brain gPPI results showing the belief-based connectivity increases between the dmPFC seed and target in Precuneus/superior parietal lobe (-24, -66, 48, k = 141, cluster-level FWE-corrected *p* = 0.0150).

Robustness Analyses 1: The effect of strategy use on behavioral and imaging results

We assessed whether participants used a strategy in the exit questionnaire used after completion of the fMRI experiment. Specifically, we asked participants whether they used a specific strategy to answer the questions. If they answered yes, we asked them in an open-ended question to describe the strategy they used. A number of participants (43%) indeed reported using some strategy when answering the questions. The most common strategy that participants reported indicates that they scanned for particular words in the vignette, such as rejected or accepted. We re-analyzed our imaging and behavioral results by adding a binary “strategy” covariate to our models. We pursued two goals in these control analyses: 1. We checked whether controlling for the strategy by including a binary covariate for strategy in our imaging and behavioral models changes the results; and 2. We checked whether there are differences across those participants that report using a strategy and those that do not at the behavioral and imaging level. Controlling for strategy does not change results at the behavioral and imaging level, with all (non-) significant effects remaining at the behavioral level, and slight changes in the extent of the activation patterns at the imaging level (see tables S1 and S2, imaging tables for models including a Strategy control regressor are largely equivalent to the ones already reported in the main paper and therefore not reported here, but are available upon request).

Additionally, we probed for behavioral and activation differences as a function of strategy. We find that at the behavioral level there are no significant interactions with our treatments for both dependent variables (accuracy and reactions time). More specifically, using a strategy did not impact the accuracy of the answers, as there were not main and interaction effects of strategy (Table S1). Reaction times were marginally faster overall for people reporting having used a strategy, but this did not impact the performance differentially across the two task domains as there were no interaction effects with strategy (life and econ, Table S2). Moreover, we find no effect of strategy in our neuroimaging results of the economic-games FBT task. In other words, we did not observe differential activations during belief relative to outcome for those subjects that reported using a strategy compared to those that did not. Interestingly, however, neuroimaging results in the standard false belief task were impacted by self-reported strategy use, indicating that strategies affect the neural correlates of the original FBT in the Putamen, anterior cingulate cortex (ACC) and regions within the insula (see Table S3).

	Strategy control			Strategy modulation		
	Chisq	Pr(>Chisq)		Chisq	Pr(>Chisq)	
Belief	17.18	<0.001	***	16.43	<0.001	***
Life	1.86	0.17		1.49	0.22	
Threat	2.36	0.12		2.38	0.12	
Strategy	0.02	0.90		0.01	0.92	
Belief x Life	18.31	<0.001	***	18.49	<0.001	***
Belief x Strategy				0.03	0.85	
Life x Strategy				1.36	0.24	
Belief x Life X Strategy				0.10	0.76	

Table S1. ANOVA tables reflecting accuracy results from mixed models including a Strategy control variable in the left columns, and strategy as a moderator variable on the right.

	Strategy control			Strategy modulation		
	Chisq	Pr(>Chisq)		Chisq	Pr(>Chisq)	
Belief	4.53	0.03	*	4.25	0.04	*
Life	64.05	<0.001	***	63.49	<0.001	***
Threat	0.49	0.48		0.49	0.48	
Strategy	3.66	0.06		3.67	0.06	
Belief x Life	276.86	<0.001	***	275.95	<0.001	***
Belief x Strategy				0.20	0.65	
Life x Strategy				0.00	0.95	
Belief x Life X Strategy				0.89	0.35	

Table S2. ANOVA tables reflecting reaction time results from mixed models including a Strategy control variable in the left columns, and strategy as a moderator variable in the right columns.

Structure	L/R	Cluster Size	x	y	z	Peak t
Economic Game						
*Vignette Period: (Belief > Outcome) * Strategy*						
No significant effects						
*Question Period: (Belief > Outcome) * Strategy*						
No significant effects						
Life Story						
*Vignette Period: (Belief > Outcome) * Strategy*						
Anterior insula	L	81	-51	5	-7	5.06
Posterior insula	L	74	-51	-13	14	4.43
Middle cingulate cortex	L	97	-12	8	41	5.27
*Question Period: (Belief > Outcome) * Strategy*						
Cerebellum	Bill.	242	3	-73	-13	4.90
Putamen	R	109	27	2	8	5.13
Putamen	L	196	-30	-16	5	4.74
Thalamus	R	86	12	-7	8	4.44
Anterior Cingulate Cortex	Bil.	102	-3	35	14	4.71
DLPFC	R	78	42	47	20	4.48
Middle Frontal Gyrus	R	80	27	38	41	4.84
Supplementary Motor Area	Bil.	105	0	-7	50	4.68

Table S3. Whole-brain analysis of the interaction between Strategy and the mentalizing effect during the economic game and life story FBT respectively ($p < 0.05$ FWE corrected at cluster-level). DLPFC: dorsolateral prefrontal cortex.

Robustness Analyses 2. The effects of game type on performance and reaction time.

We performed exploratory analyses testing whether participants performed differentially across trust game and ultimatum game trials. It is noteworthy that these analyses are exploratory, because the original analysis plan was designed to collapse across the two economic games to provide maximal power for the fMRI analyses and a minimum of 24 trials per condition as recommended for fMRI experimental designs (Delgado et al., 2005; Desmond & Glover, 2002; Huettel & McCarthy, 2001). In these analyses we focus on the economic game scenarios and, as in the previous robustness analyses reported above, we focus on the fMRI data only. We conducted a general linear mixed model following procedures outlined in the main paper. Mixed models included an additional factor for Game Type that separates ultimatum game and trust game trials. Analyses were conducted separately for accuracy and log reaction times and included Belief and Game Type as regressors, controlling for Threat as regressor of no interest. We used maximal possible models that are comparable to the ones reported in the main paper and included a random intercept for subject.

For accuracy, we find significant main effects for Belief (X2 = 8.78, $p = 0.031$) and Game Type (X2 = 8.78, $p = 0.031$; coefficients have opposite signs for each main effect). Importantly, there is a significant Belief by Game Type interaction (X2 = 15.65, $p <0.001$). Follow-up tests using the Sidak method indicate that this interaction is driven by a significant difference in performance in the trust game ($z = 5.47$, $p < 0.001$), but not in the ultimatum game ($z = -0.548$, $p = 0.58$). This difference indicates that, in the trust game, participants performed significantly better in the belief condition (percent correct = 0.99) compared to the outcome condition (percent correct = 0.895). Note, however, that performance in the trust game vignettes remains high (89.5%) in the outcome condition.

For log reaction times, we find significant main effects for Belief (X2 = 158.07, $p < 0.001$) and Game Type (X2 = 50.63, $p < 0.001$). Importantly, there is a significant Belief X Game Type interaction (X2 = 277.09, $p <0.001$). Follow-up tests using the Sidak method indicate that this interaction is driven by a crossover effect indicating significant faster performance in the belief (0.819) compared to the outcome (0.898) condition during trust games (estimate = -0.079, $t = -3.16$, $p = 0.002$), and the reverse in the ultimatum game (estimate = 0.53, $t = 21.41$, $p < 0.001$; belief average = 0.902; outcome average = 0.373).

Jointly, these results indicate faster performance specifically in the outcome condition of the UG, and slower but worse performance in the outcome condition of the TG. This compares well with the main results, such that the performance differences remain largely confined to the outcome condition when considering game types separately.

	Accuracy				Reaction Time			
	Model 1		Model 2		Model 1		Model 2	
	Chisq	Pr(>Chisq)	Chisq	Pr(>Chisq)	Chisq	Pr(>Chisq)	Chisq	Pr(>Chisq)
**Belief**	136.96	p<0.001	158.07	p<0.001	136.96	p<0.001	158.07	p<0.001
**Game Type**	50.34	p<0.001	50.63	p<0.001	50.34	p<0.001	50.63	p<0.001
**Threat**	0.05	0.82	0.04	0.85	0.05	0.82	0.04	0.85
**Belief x Game Type**			277.09	p<0.001			277.09	p<0.001
**Observations**	1765 (37)		1765 (37)		1765 (37)		1765 (37)	
**AIC**	1890.8		1615.8		1890.8		1615.8	

Table S4. ANOVA tables reflecting results from mixed models including a Game Type control variable in the left columns (Model 1), and Game Type as a moderator variable in the right columns (Model 2) for Accuracy and Reaction Time respectively.

Robustness Analyses 3. Identifying and controlling for speed accuracy trade-offs using BIS.

To further investigate the potential presence of speed-accuracy trade-offs, we therefore inspected speed accuracy trade-offs in more detail. We first computed correlations between accuracy (mean percent correct) and speed (mean RT) over the entire dataset. A mixed regression that included all trials with mean accuracy as the dependent variable and mean RT as the fixed and random effects factor, as well as subject-wise random intercepts did not reveal a significant correlation between Accuracy and RT ($p = 0.2106489$).

We repeated this exercise for each condition to test whether speed and accuracy correlate for separate cells within our experimental design. We do not find significant correlations in the economic belief [$r = -0.129$, $p = 0.274$] and the economic outcome conditions [$r = -0.182$, $p = 0.121$]. In the life belief and life outcome conditions, however, we find small to moderate correlations between speed and accuracy [life belief: $r = -0.279$, $p = 0.016$; life outcome: $r = -0.434$, $p < 0.001$]. We show these correlations in Figure S4. Note that the relationship between accuracy and RT was negative, indicating that participants who performed faster were also more accurate overall in these conditions. This is in the opposite direction of what is typically observed for speed-accuracy trade-offs (Drugowitsch et al., 2015; Palmer et al., 2005).

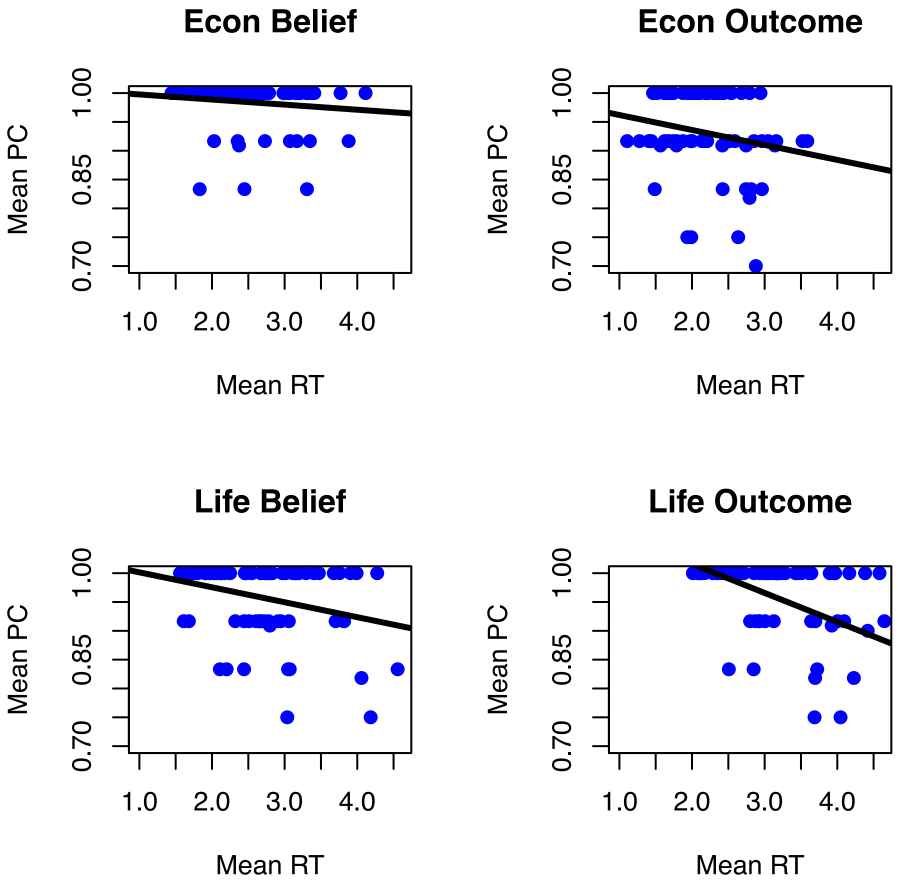

Figure S4. Correlations between speed (mean RT) and accuracy (mean PC) across the factors Life and Belief.

To correct for potential speed accuracy trade-offs found in the two Belief conditions, we performed an additional analysis using the Balanced Integration Score (BIS) as dependent variable. BIS is computed by subtracting standardized RT from the standardized percentage of the correct response, reflecting accuracy corrected for speed (Liesefeld & Janczyk, 2019). BIS scores were computed separately for each subject and for each Life and Belief condition and subsequently entered into a repeated measures ANOVA. Results yielded highly similar results to those reported in the main paper showing a main effect of Belief and a Belief x Life interaction. An additional main effect of Life is also found. Given that main effects can be largely ignored in the presence of the interaction, these results are highly similar to the ones reported in the main text, indicating that our results are likely not driven by speed-accuracy trade-offs.

	**Model 1**		**Model 2**	
	**Chisq**	**Pr(>Chisq)**	**Chisq**	**Pr(>Chisq)**
**Belief**	14.14	p<0.001	15.34	p<0.001
**Task Domain**	23.80	p<0.001	23.80	p<0.001
**Threat**	2.60	0.11	2.83	0.09
**Belief x Task Domain**			18.74	p<0.001
**Observations (N)**	296 (37)		296 (37)	
**AIC**	447.95		431.21	

Table S5. ANOVA results corrected for speed-accuracy trade-offs using BIS

Regression Equations for Behavioral Analyses

Below we report the maximal models that terminated without error in Wilkinson-Rogers notation for the main behavioral analyses reported in the paper separated by dependent variable:

1. **Accuracy Model**

accuracy ~ Belief + Task Domain + Belief * Task Domain + Shock + (1 + Task Domain | ID)

2. **Reaction Time Model**

log_rt ~ Belief + Task Domain + Belief * Task Domain + Shock + (1 + Belief * Task Domain || ID)

With accuracy coded as 0 for incorrect answers and 1 for correct answers, log RT is the logarithm of the reaction time, and Belief, Task Domain and Shock entered as factors and converted to effect coding. "||" indicates suppression of random effects correlations in reaction time models.

References

Delgado, M. R., Miller, M. M., Inati, S., & Phelps, E. A. (2005). An fMRI study of reward-related probability learning. *NeuroImage*, *24*(3), 862–873.

Desmond, J. E., & Glover, G. H. (2002). Estimating sample size in functional MRI (fMRI) neuroimaging studies: Statistical power analyses. *Journal of Neuroscience Methods*, *118*(2), 115–128.

Drugowitsch, J., Deangelis, G. C., Angelaki, D. E., & Pouget, A. (2015). Tuning the speed-accuracy trade-off to maximize reward rate in multisensory decision-making. *ELife*, *4*(JUNE2015).

Huettel, S. A., & McCarthy, G. (2001). The effects of single-trial averaging upon the spatial extent of fMRI activation. *Neuroreport*, *12*(11), 2411–2416.

Liesefeld, H. R., & Janczyk, M. (2019). Combining speed and accuracy to control for speed-accuracy trade-offs(?). *Behavior Research Methods*, *51*(1), 40–60.

Palmer, J., Huk, A. C., & Shadlen, M. N. (2005). The effect of stimulus strength on the speed and accuracy of a perceptual decision. *Journal of Vision*, *5*(5), 1–1.
